# Characterization of the transcriptome profiles related to globin gene switching during *in vitro *erythroid maturation

**DOI:** 10.1186/1471-2164-13-153

**Published:** 2012-04-26

**Authors:** Biaoru Li, Lianghao Ding, Wei Li, Michael D Story, Betty S Pace

**Affiliations:** 1 Department Pediatrics, Georgia Health Sciences University, 1120 15th St. CN-4112, Augusta, GA 30912, USA; 2 Department of Radiation Oncology, University of Texas Southwestern Medical Center, 5323 Harry Hines Blvd, Dallas, TX 75390, USA; 3 Department of Psychiatry, University of Texas Southwestern Medical Center, 5323 Harry Hines Blvd, Dallas, TX 75390, USA

**Keywords:** Gene profiling, Erythroid maturation, γ-globin, β-globin, Hemoglobin switching, Fetal hemoglobin

## Abstract

**Background:**

The fetal and adult globin genes in the human β-globin cluster on chromosome 11 are sequentially expressed to achieve normal hemoglobin switching during human development. The pharmacological induction of fetal γ-globin (*HBG*) to replace abnormal adult sickle β^S^-globin is a successful strategy to treat sickle cell disease; however the molecular mechanism of γ-gene silencing after birth is not fully understood. Therefore, we performed global gene expression profiling using primary erythroid progenitors grown from human peripheral blood mononuclear cells to characterize gene expression patterns during the γ-globin to β-globin (γ/β) switch observed throughout *in vitro *erythroid differentiation.

**Results:**

We confirmed erythroid maturation in our culture system using cell morphologic features defined by Giemsa staining and the γ/β-globin switch by reverse transcription-quantitative PCR (RT-qPCR) analysis. We observed maximal γ-globin expression at day 7 with a switch to a predominance of β-globin expression by day 28 and the γ/β-globin switch occurred around day 21. Expression patterns for transcription factors including *GATA1, GATA2, KLF1 *and *NFE2 *confirmed our system produced the expected pattern of expression based on the known function of these factors in globin gene regulation. Subsequent gene expression profiling was performed with RNA isolated from progenitors harvested at day 7, 14, 21, and 28 in culture. Three major gene profiles were generated by Principal Component Analysis (PCA). For profile-1 genes, where expression decreased from day 7 to day 28, we identified 2,102 genes down-regulated > 1.5-fold. Ingenuity pathway analysis (IPA) for profile-1 genes demonstrated involvement of the Cdc42, phospholipase C, NF-Kβ, Interleukin-4, and p38 mitogen activated protein kinase (MAPK) signaling pathways. Transcription factors known to be involved in γ-and β-globin regulation were identified.

The same approach was used to generate profile-2 genes where expression was up-regulated over 28 days in culture. IPA for the 2,437 genes with > 1.5-fold induction identified the mitotic roles of polo-like kinase, aryl hydrocarbon receptor, cell cycle control, and *ATM *(Ataxia Telangiectasia Mutated Protein) signaling pathways; transcription factors identified included *KLF1, GATA1 *and *NFE2 *among others. Finally, profile-3 was generated from 1,579 genes with maximal expression at day 21, around the time of the γ/β-globin switch. IPA identified associations with cell cycle control, ATM, and aryl hydrocarbon receptor signaling pathways.

**Conclusions:**

The transcriptome analysis completed with erythroid progenitors grown *in vitro *identified groups of genes with distinct expression profiles, which function in metabolic pathways associated with cell survival, hematopoiesis, blood cells activation, and inflammatory responses. This study represents the first report of a transcriptome analysis in human primary erythroid progenitors to identify transcription factors involved in hemoglobin switching. Our results also demonstrate that the *in vitro *liquid culture system is an excellent model to define mechanisms of global gene expression and the DNA-binding protein and signaling pathways involved in globin gene regulation.

## Background

The fetal and adult globin genes in the β-globin cluster on chromosome 11 are expressed in a stage-specific manner during development to achieve the normal γ/β-globin gene switch after birth [1-4]. A large group of hemoglobin disorders result from mutations in the β-like globin genes including sickle cell anemia caused by an A to T mutation in *HBB *(β-globin) at the sixth position to produce β^S^-globin [5]. The association of two α-globin chains with two β^S^-globin subunits forms hemoglobin S which undergoes non-covalent polymerization due to abnormal intermolecular contacts under low oxygen conditions. This produces red blood cell sickling leading to the clinical symptoms observed in sickle cell anemia [6].

Extensive research has shown the beneficial effect of γ-globin reactivation by pharmacologic methods to induce fetal hemoglobin as a treatment modality for sickle cell patients. One such drug hydroxyurea was approved in 1998 [7]. Numerous laboratories have ongoing efforts to identify additional less toxic agents that induce fetal hemoglobin however few have reach clinical trials [8,9]. Therefore defining molecular mechanisms of globin gene regulation provides an approach to define specific strategies for γ-globin gene reactivation. With the availability of high throughput genomic methods, research aimed at the discovery of global mechanisms of gene regulation using *in vitro *models is now feasible [10] to establish personalized medical therapy [11].

To date, a limited number of transcriptome profiles have been reported for global genomic analysis in human erythroid cells. For example, K562 cells induced with hemin [12] were used to characterize transcriptomes related to drug induced erythroid differentiation. Subsequently, five studies have been published to characterize gene profiling during normal human erythroid differentiation using *in vitro *liquid culture systems [13-17]. Recently, Merryweather-Clarke et al. [17] used peripheral blood mononuclear cells combined with fluorescent activated cell sorting for the CD71 and CD36 surface markers. They analyzed expression data generated from erythroid progenitors isolate at the various stages of erythropoiesis and identified proteins with undiscovered function in erythroblast. Collectively, these studies generated significant findings regarding the erythroid transcriptome however our study is the first to characterize the transcriptome associated with the γ/β-globin switch.

To achieve this end, microarray analysis was performed using the Illumina whole genome platform to define global gene expression patterns associated with the γ/β-globin switch during primary erythroid maturation. We observed maximal γ-globin and β-globin gene expression at day 7 and 28 respectively with the γ/β-globin switch occurring around day 21. We defined three major gene profiles consistent with a potential role of γ-globin activator (profile-1) with gene silencing from day 7 to day 28; β-globin activator (profile-2) showing increased gene transcription from day 7 to day 28 and profile-3 genes defined by maximal expression at day 21 when the γ/β-globin switch was observed. Using > 1.5-fold change in expression, we identified 2,102 profile-1 genes some of which were involved in cell signaling through the p38 and ERK MAPK and erythropoietin receptor pathways. Another 2,437 profile-2 genes with patterns consistent with a positive role in β-globin regulation and 1,579 profile-3 genes that might play a role in the γ/β-globin switch were identified. A global approach to define the transcriptome involved in globin gene expression during erythropoiesis can be used to generate testable hypotheses of γ-globin regulation and the development of strategies for fetal hemoglobin induction to treat sickle cell anemia.

## Results and discussions

### The γ/β-globin switch is observed in the one-phase liquid culture system

**Figure 1:**
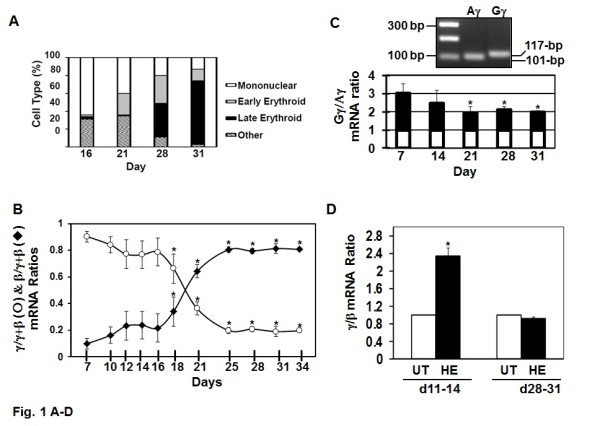
**The γ/β-globin switch is recapitulated in the one-phase liquid culture system**. Cells grown in the one-phase system were harvested every 2-3 days and analyzed as follows (see Methods). A) Cell morphology was determined by Giemsa stain and different cell types classified based on standard morphologic features. B) RT-qPCR analysis was performed to determine changes in γ-globin and β-globin mRNA levels during erythroid maturation. C) RT-qPCR was performed with Gγ and Aγ specific primers to determine the Gγ:Aγ mRNA ratio during erythropoiesis. Aγ-globin expression was normalized to one (white bars) and the relative changes in Gγ-globin mRNA are shown in the black bars. D) Progenitors were induced on day 11 or day 28 with 50 μM hemin (HE) for 72 hrs and then RT-qPCR was completed.

To study global mechanisms of globin gene regulation, we established the *in vitro *one-phase liquid culture system developed by Uddin et al. [18]. We modified this system using, human peripheral blood mononuclear cells grown in the presence of stem cell factor (50 ng/mL), Interleukin-3 (50 ng/mL), and erythropoietin 4 IU/mL from day 0. Cells were harvested every two to three days for cell morphology by Giemsa stain and γ-globin and β-globin gene mRNA levels were quantified by RT-qPCR analysis (see Methods). From day 16 to day 31, the percentage of early and late erythroid progenitors increased from 5% to 84% (Figure 1A). Early erythroid cells possess a deep blue cytoplasm consistent with basophilic erythroblasts followed by maturation into late orthochromatophilic erythroblasts by day 28 (data not shown). During the same time period γ-globin and β-globin gene expression was monitored by RT-qPCR. We observed progressive γ-globin silencing and concomitant β-globin activation with the γ/β-globin switch occurring around day 21 (Figure 1B) recapitulating human hemoglobin switching observed during development.

We next analyzed the response of the γ-globin gene in more detail to establish our system as a good model for performing transcriptome analysis. At birth, fetal hemoglobin composes 80-90% of the total hemoglobin synthesized and it gradually decreases to < 1% by 10 months in normal infants [19]. Fetal hemoglobin is a heterogeneous mixture of γ-globin polypeptide chains containing either glycine (Gγ) or alanine (Aγ) at residue 136 [20]. At birth Gγ-chains predominate, however, a switch to predominantly Aγ-chains arises during the first year of life going from a 3:1 to 1:1 Gγ:Aγ ratio. As shown in Figure 1C, the ratio of Gγ:Aγ-globin expression changed from 2:1 at day 7, to 1:1 by day 31 a pattern similar to that observed after birth [21].

Subsequently, we tested the ability to activate γ-globin expression in our human erythroid culture system using hemin, a known fetal hemoglobin inducer. Hemin activated γ-globin 2.3-fold at day 11 in contrast to a lack of induction at day 28 (Figure 1D) suggesting the ability of hemin to further enhance a transciptionally active γ-globin gene at day 11. By contrast, day 28 cultured cells carrying a silenced γ-globin gene were resistant to induction by hemin. These data established our one-phase liquid culture as a model to study erythropoiesis that can be used to define the erythroid transcriptome associated with the γ/β-globin switch.

### Data mining approach

**Figure 2:**
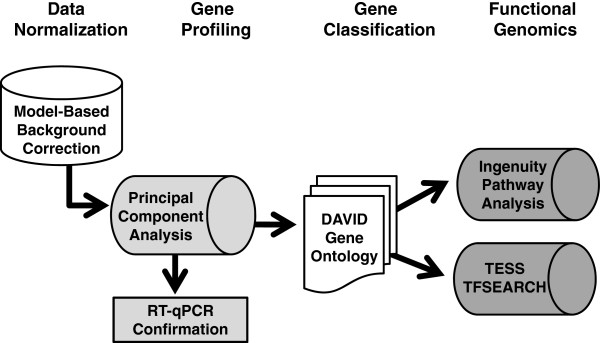
**Data mining strategy**. Shown is a schematic diagram outlining our genomic bioinformatics approach for the data generated by microarray analysis.

Based on the timing of the γ/β-globin gene switch, we performed microarray analysis to define the erythroid cells transcriptome. Our data mining approach (Figure 2) consisted of four steps: 1) data normalization after microarray chip hybridization using Model-Based Background Correction (MBCB) for BeadArrays and quartile normalization; 2) time-course gene expression profiling using PCA and the confirmation of gene profiles for a subset of genes by RT-qPCR analysis; 3) gene classification using DAVID (the Database for Annotation, Visualization and Integration Discovery) gene ontogeny (GO); and 4) functional genomics to define the signaling pathways potentially involved in globin regulation using Ingenuity Pathway Analysis (IPA). *In silico *analysis with Transcription Element Search Site (TESS), TFSEARCH, Weeder H, and FIRE platforms was also performed to identify transcription factors with predicted binding sites in the β-globin locus.

### Data collection and gene expression profiling

We collected three RNA samples at day 7, 14, 21, and 28 which allowed us to perform gene expression profiling over a time course. The advantage of this approach is the use of well-established time-course algorithms for data mining. However, the variability that can occur at different times in culture can introduce errors in gene expression data, but replicate samples help to address this concern. RNA quality check was performed prior to microarray analysis on the Illumina HumanWG-6V2 Expression BeadChip containing 48,700 probes on the Bio-Rad *Experion *system. Automated electrophoresis qualities confirmed RQI (RNA Quality Index) values > 8.0 for all samples included in our microarray analysis (Additional file 1: Figure S1). The raw data generated are summarized in Additional file 2: Table S1, Additional file 3: Table S2, Additional file 4: Table S3, Additional file 5: Table S4.

**Figure 3:**
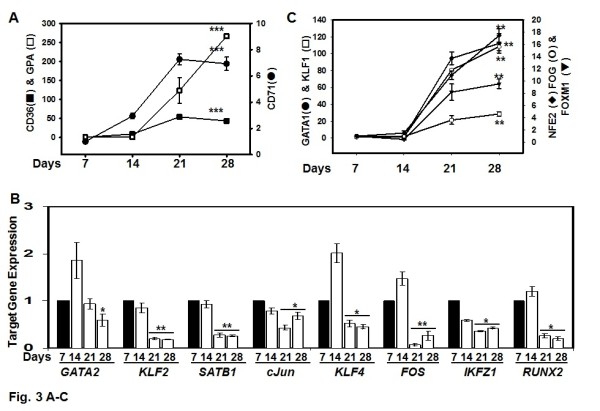
**Gene expression analysis of transcription factors known to regulate globin gene expression**. A) Gene expression patterns for established erythroid markers are shown. Note the different scales for the left and right Y-axis to reflect the magnitude of difference in gene expression. Shown is the mean ± SEM; *** = *p *< 0.001. B) Time-course curves of genes previously reported to be involved in γ-globin regulation are shown; * = *p *< 0.05; ** = *p *< 0.01. C) Graphed is the expression profiles for genes known to regulate β-globin expression. The raw data for the genes shown are included in Additional file 2: Table S1, Additional file 3: Table S2, Additional file 4: Table S3, Additional file 5: Table S4.

After the raw data was normalized several gene probes including *S18, S28, DDX5 *and *ACTB *were used to determine data consistency. The results show no significant differences (*p *> 0.05) in expression across all microarrays for the internal control genes. We also observed that the expression of erythroid cell biomarkers such as CD36 and glycophorin A (GPA) significantly increased (44-fold to 266-fold) from day 7 to day 28 (*p *< 0.001) (Figure 3A); however CD71 levels increased 7-fold (*p *< 0.001) followed by slightly decreased expression by day 28. These findings are consistent with the expression patterns of cell surface markers during normal human erythroid differentiation [22,23].

We next examined the expression profiles of genes known to be involved in γ-globin and β-globin regulation prior to global data mining. The following genes including *GATA2 *[24], *cJun *[25], *KLF2 *[26], *SATB1 *[27], *KLF4 *[28], *FOS *[29], *IKZF1 *[30] and *RUNX2 *[31], which have been reported to be associated with γ-globin regulation were analyzed (Figure 3B). Simultaneous with γ-globin silencing, we observed decreased expression from day 7 to day 28 for *GATA-1, KLF2, KLF4, FOS, SABT1, cJun, IKFZ1 *and *RUNX2 *among others in agreement with published data.

Genes previously shown to be associated with β-globin regulation including *KLF1 *[32], *GATA1 *[33], *NFE2 *[34], *FOG1 *[35] and *FOXM1 *[16] were examined as well. We observed significant *KLF1 *(108-fold; *p *< 0.01) and *GATA1 *(112-fold) induction compared to 17.5-fold induction of *NFE2, FOG1 *(4.6-fold) and *FOXM1 *(9.6-fold) from day 7 to day 28, findings consistent with published results (Figure 3C). These data support our line of reasoning that whole genome gene profiling might identify other transcription factors involved in globin gene regulation.

Before data mining, the normalized microarray data was examined in a multiple regression algorithm to study chip-chip reproducibility (Additional file 1: Figure S4A). For the three chips analyzed at day 7 the adjusted R^2 ^= 0.99 (F-value 4.94E + 0.6 and P-value 2.2E-16), for day 14 triplicates the adjusted R^2 ^= 0.98 (F-value 2.96E + 0.6 and P-value 2.2E-16), for day 21 chips, the adjusted R^2 ^= 0.99 (F-value 3.51E + 0.6 and P-value 2.2E-16), and for day 28 triplicates the adjusted R^2 ^= 0.99 (F-value 4.92E + 0.6 and P-value 2.2E-16). These data demonstrate a high correlation of data reproducibility between microarray chips.

Initial gene profiling studies were conducted by Principal Component Analysis (PCA), which is an approach to identify patterns in large datasets. PCA involves a multivariate mathematical procedure that transforms a number of correlated variables into a smaller number of uncorrelated variables called principal components. The first principal component accounts for as much of the variability in the dataset as possible, and each succeeding component accounts for the remaining variability. Normalized data were used for PCA to define three major expression profiles including genes silenced from day 7 to day 28 (profile-1), genes activated over the culture period (profile-2) and genes with maximal expression at day 21 (profile-3) during the time of the γ/β-globin switch.

**Figure 4:**
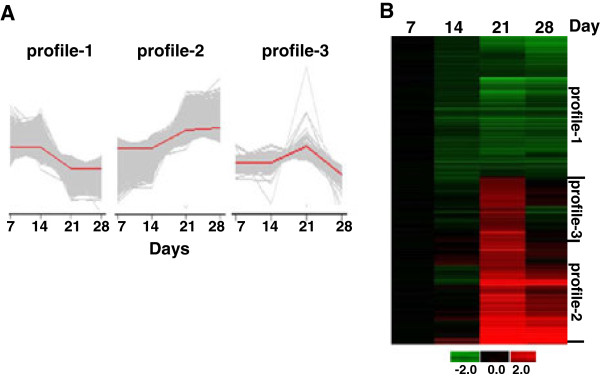
**Principal component analysis (PCA)**. A) PCA was performed for gene subsets with > 1.5-fold change in expression over the 28-day culture period (See Methods). The results are shown for the three major gene profiles generated by PCA. The red line represents the mean value of time-course changes (days 7, 14, 21 and 28). B) Expression values were normalized to the mean values of Day 7 samples. The resultant log_2 _ratios at each day were averaged and displayed in the heatmap. The color bar indicates log_2 _ratio of change in gene expression.

Because our genomics data were mined to follow the expression patterns of the γ- and β-globin genes patterns, we chose a statistical analysis consisting of F-distribution with the Analysis of Variance (ANOVA) value = 0.01 and False Discovery Rate (FDR) = 0.05. Our goal was to maintain globin gene expression profiles therefore permutation analysis was not conducted. To establish gene subsets for our subsequent functional genomics analysis, we identified gene with > 1.25-, > 1.5- and > 2-fold change in expression from day 7 to day 28 by PCA (Table 1). For the highest stringency of 2-fold, we observed 492, 357, and 325 genes for profile-1, -2 and -3 respectively. The PCA data for genes with > 1.5-fold differential expression are summarized in Additional file 6: Table S5; these gene were used for the hierarchical cluster studies shown in Figure 4B which further illustrates the three major gene profiles consistent with the PCA data.

**Table 1:** Summary of Principle Component Analysis

	> 1.25-fold	> 1.5-fold*	> 2-fold
profile-1	3,317	1,610	492

profile-2	3,105	2,080	357

profile-3	2,120	1,254	325

*Genes with > 1.5-fold change were used for the subsequent bioinformatics analyses

It is established practice to confirm the expression pattern obtained by microarray with RT-qPCR analysis. Therefore, we confirmed the microarray data for 37 experimental genes chosen from profile-1, profile-2, and internal controls. As shown in Table 2, 93.5% (29 out of 31) of gene expression patterns identified by microarray analysis were confirmed by RT-qPCR. Of note are similar patterns for the hematopoietic transcription factors *KLF1, NFE2 *and *GATA1 *however the magnitude of change was different. To further validate our microarray data, regression analysis was completed for the two data sets. Our results showed that gene expression patterns obtained by RT-qPCR analysis correlated to that observed by microarray assay. For example, profile-1 genes including *GATA2 *showed R^2 ^= 0.80, *p *= 0.104 and for *SATB1 *R^2 ^= 0.977, *p *= 0.012. The same analysis for profile-2 genes showed *KLF1 *with a R^2 ^= 0.91, *p *= 0.045, *GATA1 *(R^2 ^= 0.99, *p *= 0.003), *NFE2 *(R^2 ^= 0.755, *p *= 0.131) and *FOG1 *(R^2 ^= 1, *p *= 0.00). However, not all fold change in gene expression correlate between the two techniques which have been reported by other investigators [36,37]. In our study, *c-Myc*, and *18S *show disparate results that might reflect differences in sensitivity of the two techniques. Inconsistencies for 18S rRNA expression due to technical issue such as mRNA degradation have been reported [38]. Overall, we observed a high correlation between data generated by microarray and RT-qPCR to support the accuracy of our gene profiling approach.

**Table 2:** Comparison between microarray and RT-qPCR mRNA levels

Number	Gene sources	Symbol	Microarray(Mean fold change)				RT-qPCR(Mean fold change)			
			
			D7	D14	D21	D28	D7	D14	D21	D28
1	profile-1	*STAT5A*	1.00	0.50	0.99	0.35	1.00	1.18	0.56	0.46
2	profile-1	*DBP*	1.00	1.13	0.28	0.67	1.00	0.08	0.28	0.48
3	profile-1	*FOXQ1*	1.00	0.66	0.57	0.54	1.00	0.00	0.05	0.03
4	profile-1	*NFIL3*	1.00	0.75	0.23	0.19	1.00	0.10	0.35	0.26
5	profile-1	*MTF1*	1.00	0.66	0.50	0.75	1.00	0.58	0.97	0.82
6	profile-1	*JUN*	1.00	0.79	0.43	0.68	1.00	0.05	1.10	0.22
7	profile-1	*KLF2*	1.00	0.86	0.20	0.18	1.00	0.08	0.11	0.11
8	profile-1	*CEBPA*	1.00	1.20	0.09	0.10	1.00	0.02	0.18	0.17
9	profile-1	*CREBBP*	1.00	0.85	0.70	0.99	1.00	0.81	0.65	0.61
10	profile-1	*SP3*	1.00	0.84	0.88	0.87	1.00	0.98	0.85	0.47
11	profile-1	*SATB1*	1.00	0.94	0.28	0.26	1.00	0.80	0.24	0.15
12	profile-1	*c-Myc*	1.00	0.95	1.05	0.62	1.00	11.9	14.63	11.95
13	profile-1	*GATA2*	1.00	1.87	0.95	0.59	1.00	4.00	1.20	1.20
14	profile-1	*KLF11*	1.00	1.22	0.58	0.70	1.00	2.16	0.58	0.70
15	profile-2	*FOG1*	1.00	0.96	3.64	4.63	1.00	0.99	3.70	4.70
16	profile-2	*USF1*	1.00	1.01	0.85	1.26	1.00	0.71	1.45	2.02
17	profile-2	*SP1*	1.00	0.86	1.20	1.55	1.00	1.16	1.02	1.39
18	profile-2	*KLF3*	1.00	0.95	1.45	1.86	1.00	0.95	1.83	1.72
19	profile-2	*KLF1*	1.00	1.75	80.72	108.50	1.00	2.22	6.70	13.80
20	profile-2	*POU2F1*	1.00	0.91	1.28	1.90	1.00	0.57	1.23	1.16
21	profile-2	*KLF13*	1.00	0.98	1.12	1.83	1.00	2.16	0.58	8.70
22	profile-2	*GATA1*	1.00	1.83	94.63	112.31	1.00	1.80	7.40	8.40
23	profile-2	*NF-E2*	1.00	1.53	10.98	17.47	1.00	17.6	85.80	776.40
24	profile-2	*ATF2*	1.00	1.25	1.69	2.03	1.00	2.70	1.50	2.40
25	profile-2	*FGFR3*	1.00	1.20	47.87	39.77	1.00	8.22	956.	923.45
26	profile-2	*MAPKK6*	1.00	1.01	1.65	2.07	1.00	1.39	1.13	2.12
27	profile-2	*HSP90kDa*	1.00	0.95	1.65	1.75	1.00	0.63	1.50	8.57
28	profile-2	*v-Myc*	1.00	1.03	1.70	2.49	1.00	30.5	0.45	2.44
29	profile-2	*CUTL1*	1.00	1.02	1.58	2.20	1.00	1.02	0.42	2.06
30	Reference 1	*ACTB*	1.00	1.05	0.84	0.91	1.00	1.00	1.00	1.00
31	Reference 2	*18 S*	1.00	0.96	1.00	1.06	1.00	0.68	1.62	1.41

### DAVID gene ontogeny (GO) gene function analysis

To properly classify gene function, the transcriptome generated from microarray analysis was analyzed by GO software to: a) address whether or not the data mining correctly identified related genes expressed in the expected cell type using the Kappa score; b) define functional annotation and transcriptome categories based on biological processes, cellular components and molecular function to address the enriched relationships among many genes; and c) reflect the individual GO functional level by over-representation with higher numbers of genes and significant p-values in the GO terms to determine and classify GO groups [39,40]. We completed a GO clustering analysis for each gene profile subset with > 1.5-fold change in expression during erythroid maturation using the DAVID platform [41]. For interpretation of our analysis, the higher the Kappa score for a given genomic profile, the stronger the agreement with the cell type from which the specimens were extracted; if Kappa = 1, then there is perfect agreement for cell type. The detailed results and corresponding p values for each GO term are shown in Additional file 7: Table S6.

For profile-1 genes, Kappa = 1 was obtained for the GO term hematopoiesis (Enrichment score 15.91, *p *= 7.7E-10). Profile 2 genes were highly associated with the GO term erythrocyte differentiation, Kappa = 1 (Enrichment score 2.5, *p *= 1.5E-4). On the other hand, profile-3 genes had a Kappa = 1 for the GO term macromolecular complex subunit organization (Enrichment score 5.27, *p *= 3.8E-7). This latter term describes a process by which macromolecules aggregate, or disaggregate to reform, disassembly, or alter macromolecular complexes. Reorganization of these complexes has been largely reported in protein expression changes and gene switching produced in cells infected with viruses, bacteria and parasite [42]. We speculate that macromolecule complex reorganization may occur during the γ/β-globin switch.

To further characterize the genes identified in each profile we used over-representation to classify GO groups. In this analysis we investigated two major GO categories where 1) biological processes with 30 subgroups and 2) molecular function with 20 subgroups were identified (*p *< 0.05). For biological processes, the following subgroups are highlighted for profile-1: 47 genes were over-represented in hemopoiesis (Enrichment score 15.91, *p *= 7.7E-10) and 85 genes in cell activity (Enrichment score 15.91, *p *= 1.4E-29). We also observed 121 profile-2 genes related to cellular proliferation (Enrichment score 25.4, *p *= 1.43E-32) and 8 genes related to heme biosynthesis (Enrichment score 3.27, *p *= 6.5E-6). Finally, 81 genes in profile-3 were associated with nucleotide metabolism and DNA processing (Enrichment score 5.14, *p *= 1.1E-6). The GO subgroups identified would be predicted since erythropoiesis involves actively dividing hematopoietic cells, which require heme biosynthesis for normal hemoglobin production.

For the second GO category molecular function, profile-1 genes were over-represented in the hydrolase category including protein tyrosine phosphatase, MAP kinase phosphatase, GTP cyclohydrolase and tyrosine/serine/threonine phosphatase. By contrast, molecular function GO terms for profile-2 genes included iron, heme and oxygen binding proteins; profile-3 genes were related to adenyl nucleotide, ATP and nucleoside binding proteins. In summary, DAVID GO data mining classified profile-1 genes as associated with hematopoiesis while profile-2 genes were related to cell proliferation and erythrocyte differentiation. Finally, profile-3 genes were associated with alteration of a macromolecular complex, or protein switching processes requiring DNA synthesis.

### Functional genomics - Ingenuity Pathway Analysis (IPA)

IPA is software that helps researchers model, analyze, and understand complex systems by integrating data from a variety of experimental platforms and providing insight into molecular and chemical interactions, cellular phenotypes, and disease processes. We performed IPA to identify pathways involved in erythropoiesis defined at *p *< 0.05 and IPA significance value > 1.3.

**Figure 5:**
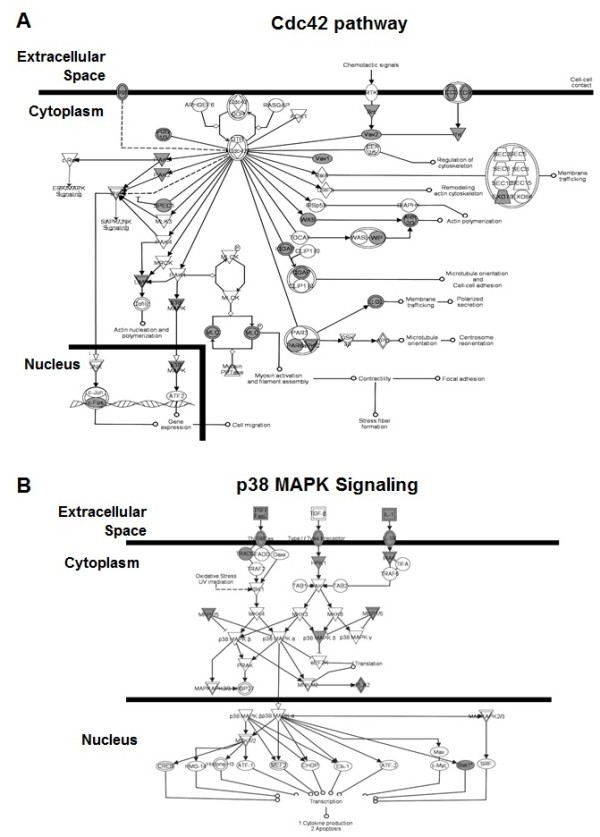
**Pathways identified by IPA**. Profile-1 genes were used for IPA. The A) Cdc42 and B) p38 MAPK signaling pathways identified for profile-1 genes are shown. The shaded symbols represent gene hits in the pathway and clear symbols representing other known genes in the signaling pathway (squares = cytokines, oval shape = transcription factors and triangle shape = kinases).

The IPA for profile-1 genes with > 1.5-fold decreased expression over 28 days is shown in Table 3. The most highly correlated pathways included Cdc42 (cell division cycle 42, GTP binding protein) (36 hits, *p *= 14.2), NF-Kβ (33 hits, *p *= 7.92), Phospholipase C (42 hit, *p *= 7.13), Interleukin 4 (17 hits, *p *= 4.78), LXR/RXR (15 hits, *p *= 3.63) and erythropoietin (14 hits, *p *= 3.17). Interestingly, the *Cdc42 *gene is not differentially expressed by microarray and RT-qPCR data (Additional file 2: Table S1, Additional file 3: Table S2, Additional file 4: Table S3, Additional file 5: Table S4 and Table 2 respectively). Although mRNA levels may not be altered, Cdc42 signaling can be activated by phosphorylation at serine 71 [43] with subsequent activation of downstream effector molecules. The latter proteins such as *PAK2 *or *SPEC1 *may be regulated at the transcriptional and posttranslational levels (Figure 5A) to explain the high score obtained for the Cdc42 pathway.

**Table 3:** Main pathways identified by profile-1 genes

Ingenuity Canonical Pathways	gene hits	-log (p-value)
Cdc42	36	14.2
Phospholipase C	42	7.13
NF-κβ	33	7.92
Interleukin-4	17	4.78
LXR/RXR Activation	15	3.63
Erythropoietin	14	3.17
PPARα/RXRα ± Activation	21	2.19
Rac	16	2.17
SAPK/JNK	14	1.92
p70S6K	17	1.88
Acute Phase Response	21	1.66
Notch	7	1.64
PPAR	12	1.56
ERK/MAPK	21	1.44
p38 MAPK	13	1.44
Insulin Receptor	16	1.43

Although most of these pathways have not been directly associated with mechanisms of globin gene regulation, there are multiple studies to support the involvement of p38 MAPK signaling in erythropoiesis (Figure 5B). For instance by IPA, p38 MAPK is activated by phospholipase C [44], Cdc42 [45], and LXR/RXR [46] signaling. Although initially identified as a protein kinase activated by stress, p38 MAPK signaling coordinates cellular responses during erythropoiesis [47]. For example, p38 MAPK is essential for the synthesis of hemoglobin [48] and p38α^-/-^mice exhibit severe anemia and die *in utero *owing to defects in angiogenesis, and placental insufficiency [49]. Furthermore, the proliferation and differentiation of erythroid progenitors is controlled by erythropoietin through the p38 MAPK pathway [50] and p38 is required for erythropoietin mRNA stability [51].

Previous data from our laboratory demonstrated that p38 MAPK signaling plays an important role in drug-mediated fetal hemoglobin induction in primary erythroid cells [52]. Moreover, *ATF2, CREBBP *and *cJun *are required to induce γ-globin expression via p38 activation. The global pathway analysis from profile-1 genes suggests that p38 MAPK plays a direct role in γ-globin gene regulation independent of drug induction. These data are in agreement with our recent publication demonstrating that p38 is required for steady-state γ-globin activation during normal erythropoiesis [53]. The specific role of other signaling pathways in globin gene regulation remains to be defined.

In contrast to efforts aimed at understanding mechanisms of γ-globin gene expression, fewer studies have focused on β-globin regulation via cell signaling pathways. In our pathway analysis of profile-2 genes, we identified the Mitotic Roles of Polo-Like Kinase (PLK) pathway (19 hits, *p *= 7.22) highly associated with β-globin activation (Table 4 and Additional file 1: Figure S5C). Although there is no direct evidence for PLK involvement in globin gene regulation, it is known that PLK can inhibit histone deacetylase 3 activity [54]. Furthermore, histone deacetylase 3 can inhibit the TATA box and produce positive regulation of β-globin expression by enhanced *USF *and *TFII *binding [55]. On the contrary, Perrine and colleagues demonstrated γ-globin gene silencing mediated by a HDAC3-NCoR repressor complex [56]. These finding suggest some protein may play a dual role in globin gene regulation.

**Table 4:** Main pathways identified by profile-2 genes

Ingenuity Canonical Pathways	gene hits	-log(p-value)
Mitotic Roles of Polo-Like Kinase	19	7.22
CHK Proteins in Cell Cycle Checkpoint Control	14	5.96
Cell Cycle: G2/M DNA Damage Checkpoint Regulation	13	5.02
Aryl Hydrocarbon Receptor	24	3.74
ATM	13	3.66
Cell Cycle: G1/S Checkpoint Regulation	11	2.34
p53	15	2.04
RAC	16	1.90
Cell Cycle Regulation by BTG Family Proteins	7	1.62

Numerous other signaling pathways including the Aryl hydrocarbon receptor, ATM, cell cycle regulation, P53 protein and RAC are activated during adult stage β-globin gene expression [57,58]. Our studies for profile-2 genes (Table 4) revealed the cell cycle control proteins, and Aryl hydrocarbon receptor, and ATM pathways with the highest number of gene hits. Of interest is the ATM pathway (Additional file 1: Figure S5D) which phosphorylates several proteins to activate the DNA damage checkpoint, leading to cell cycle arrest, apoptosis and cell differentiation [59]. This pathway also regulates DNAPK which has been associated with bone marrow failure [60]. Recent studies support a role for ATM in the regulation of erythropoiesis and β-globin expression however the exact mechanism remains unclear [61]. ATM was also identified with profile-3 genes (Table 5) suggesting this signaling pathway may play a more general role in erythroid maturation.

**Table 5:** Main pathways identified by profile-3 genes

Ingenuity Canonical Pathways	gene hits	-log(p-value)
Role of CHK Proteins in Cell Cycle Checkpoint Control	10	4.98
ATM	10	3.17
Cell Cycle: G1/S Checkpoint Regulation	9	2.40
Cell Cycle: G2/M DNA Damage Checkpoint Regulation	7	2.15
Aryl Hydrocarbon Receptor Signaling	15	2.02
RAN	4	1.91
LPS/IL-1 Mediated Inhibition of RXR Function	19	1.86
Nucleotide Excision Repair Pathway	6	1.81
Fructose and Mannose Metabolism	9	1.34

Published data to define the erythroid transcriptome in human primary progenitors [13-17] have mainly been established using a variety of liquid culture systems and a range of 0-43% differentially expressed genes have been reported. Given the differences in experimental design for the various studies it is difficult to directly compare results. The most recent study by Merryweather-Clarke et al. [17] is most similar with our design except they used the two-phase liquid culture design established by Fibach et al. [62] and a FACS sorting step to isolate purified erythroid progenitors for gene profiling. They reported the highest level of differentially expressed genes at 47% and identified potential new targets involved in erythroid maturation. By comparison, we identified 38% differentially expressed genes and identified similar cellular pathways involved in erythroid maturation by IPA and DAVID GO analysis. Our study is unique in that none of the published studies performed *in silico *transcription factor binding analysis based on major gene profiles to gain insights into the γ/β globin switching described below.

### Functional genomics - identification of β-locus transcription factor binding sites

**Figure 6:**
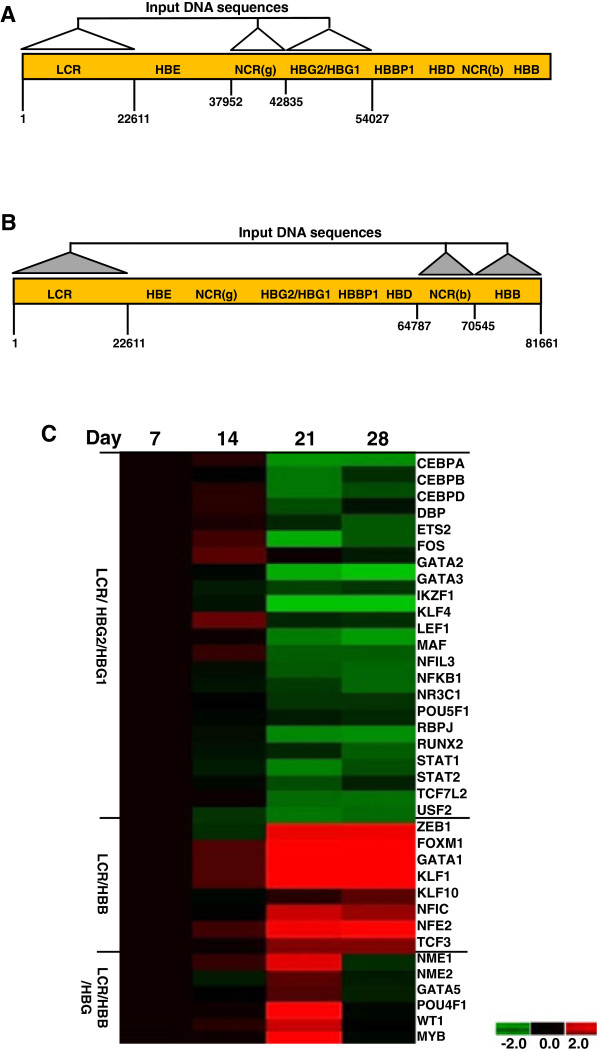
**Identification of transcription factor binding sites in the β-locus**. A) Shown is a schematic diagram of the DNA sequence positions (NG_000007.3) used in the TESS, TFSEARCH, Weeder H and Fire analysis for profile-1 genes. The figure is not drawn to scale. Abbreviations: LCR, locus control region; NCR(g), noncoding region *HBG2*; NCR(b), noncoding region *HBB*. See methods for more details of DNA sequence location. B) Shown is a schematic diagram of the genomic locations used in the transcription factor binding site analysis for profile-2 genes. Binding sites for profile-3 genes were identified using a combination of panels A and B sequences (LCR, NCR(g), *HBG2/HBG1*, NCR(b), and *HBB *regions). C) Hierarchical clustering analysis was performed for profile-1, -2 and -3 genes predicted to have binding sites in the β-locus. The data were analyzed as described in Figure 4B. The transcription factors are labeled on the right side of the image.

In the final set of analyses our goal was to identify transcription factor binding sites for profile-1, -2, and -3 proteins identified by PCA. The regions shown in Figure 6A and 6B were chosen based on the speculation that profile-1 genes (silenced from day 7 to day 28) might be trans-activators of γ-globin expression since they are expressed in a similar pattern as other factors demonstrated to activate γ-globin. Likewise, profile-2 genes (activated from day 7 to day 28) might be involved in β-globin regulation. The alternative possibility that profile-1 and profile-2 genes are β- or γ-globin silencers respectively was not tested. With this goal in mind, we performed *in silico *analysis using TESS, TFSEARCH, Weeder H, and FIRE platforms and DNA sequences from the Human Genome (Hg19 version; NG_00007.3) to identify predicted binding sites in the β-locus. Shown in Figure 6 are schematics of the regions targeted for these analyses. We examined the locus control region (LCR) consisting of five developmentally stable DNaseI hypersensitive sites (HSs) of which HS1 to HS4 are erythroid-specific [63]. This regulatory element is known to bind multiple erythroid and ubiquitous transcription factors and to mediate a critical enhancer function required for developmentally regulated globin gene expression. A unique property of the LCR is its ability to confer integration position-independent expression on a linked gene, however only HS2 act as a classical enhancer element [3]. In addition to the LCR, we tested gene-specific regions including approximately 5000 bp upstream of the *HBG2 *capsite, to 5000 bp downstream of the *HBG1 *gene (Figure 6A). These regions were investigated for binding sites of protein-1 genes silenced > 1.5-fold from day 7 to day 28.

We identified 22 transcription factors with potential binding sites in the LCR and γ-globin gene regions. For example, the GATA factors (*GATA1, 2 *and *3*) which are essential for normal hematopoiesis. *GATA2 *expression overlaps with *GATA1 *during erythroid, megakaryocytic, eosinophilic and mast cells development [64]. Consistent with our prediction model for profile-1 genes (Additional file 8: Table S7), *GATA2 *over expression in K562 cells mediates activation of the erythroid-specific genes α- and γ-globin [65]. In committed erythroid progenitors, *GATA2 *is replaced by *GATA1*. Both GATA factors co-exist in various protein complexes such as SCL/LMO2/E2A/Ldb-1 and *NFE2 *which have been demonstrated to bind directly to globin gene promoters [66]. However, *GATA3 *is expressed mainly in T-cells [64] and a direct role in globin regulation has not been documented. Other factors such as *C/EBPA*, which binds as a homodimer to certain promoters, can also forms heterodimers with the related proteins *C/EBPB *[67]. These proteins have been implicated as positive regulators of γ-globin through the distal CCAAT box.

In the DNA-binding site studies we also identified the signal transducers and activators of transcription (Stat) proteins. *Stat1 *and *Stat2 *form heterodimers in response to erythropoietin stimulation, however, others have reported that *Stat1α *and *Stat3 *can act as negative regulators in erythropoietin-induced erythroid differentiation [68]. Our laboratory demonstrated a negative role for *Stat3β *in γ-globin expression while *Stat3α *played a positive role [69]. Subsequent studies from our group demonstrated that Stat3 competes with *GATA1 *binding to an element located between +9 to +16 in the γ-globin gene 5' untranslated region [70].

The subsequent *in silico *analysis for profile-2 genes was conducted with sequences in the LCR and about 5700 bp upstream of *HBB *to the end of the β-locus (Figure 6B and Additional file 9: Table S8). Several factors known to be involved in hematopoiesis were identified such as the lineage-restricted transcription factors *GATA1, SCL/Tal1, LMO2, LDB1 *and *KLF1 *[71]. As expected, we identified *KLF1 *the most extensively characterized regulator of *HBB *expression and erythrocyte development [32]. It is a zinc finger protein that recognizes a subset of CACC motifs and acts primarily as a transcriptional activator [72]. Recent data demonstrated a role for *KLF1 *in *BCL11A *activation to produce γ-globin silencing [73]. Interestingly, our microarray data show a 20.6-fold increase in BCL11A expression from day 7 to day 28 (Additional file 6: Table S5) which is consistent with its demonstrated role as a γ-globin silencer [74-76]. In the β-locus, BCL11A is bound in HS3, approximately 3 kb downstream of *HBG1 *and upstream of *HBD *[74]. The specificity of the BCL11A binding site 5' CCAC(c/g) is variable [6]. In our *in silico *analysis, BCL11A was not predicted to bind the LCR or downstream *HBG1 *regions. This may be due to variations in the consensus binding sites and/or differences in the software algorithms used in the four platforms to identify binding sites. Our design did not include the *HBD *region and only transcription factors with predicted binding sites in all programs were retained on the list. Alternatively, BCL11A may not directly bind in the β-locus rather produce its effect by protein-protein interactions. This mechanism is supported by the ability of BCL11A to interact with GATA-1, HDAC1 and HDAC2 [74] and Sox 6 [77] among other proteins. On the other hand, we identified the known NFE2 binding sites in HS2; *NFE2 *recognizes the TCAT/C sequence of the AP-1-like core palindrome sequence present in a number of erythroid genes. *NFE2 *also has been shown to play an important role in β-globin gene regulation [78].

Finally, for profile-3 genes we analyzed all regions described for profile-1 and -2 genes reasoning that factors associate with the γ/β switch might bind throughout the β-locus. We identified six transcription factors (Additional file 10: Table S9) with predicted binding sites upstream of HS1 (NME1), in HS3 (NME2), both γ-globin genes (POU4F1), 5' of β-globin (MYB and GATA5) and in 5' HS3 (WT1). One of the most important regulators of mammalian hematopoiesis is c-MYB, an evolutionarily conserved transcription factor [79] highly expressed in immature hematopoietic cells and down-regulated during differentiation. We observed peak expression around day 21 consistent with published data. Bianchi and colleagues [80] demonstrated that c-MYB silencing in CD34^+ ^stem cells increased commitment toward the macrophage and megakaryocyte lineages, whereas erythroid differentiation was impaired. Furthermore, gene expression profiling analysis identified KLF1 and LIM Domain Only 2 as putative targets, which could account for the effects of c-MYB knockdown. A genome-wide association study has shown polymorphisms in the *HBS1L-MYB *intergenic (HMIP) region are highly associated with elevated fetal hemoglobin levels in Chinese β-thalassemia heterozygotes [81]. The exact mechanism by which these variants result in elevated fetal hemoglobin remains unclear, although it has been suggested MYB may mediate this effect [82].

The Wilms' tumor protein Wt1 is another interesting profile-3 gene with predicted binding in the β-locus. It is required for embryonic development and has been implicated in hematologic disorders [83]. Wt1 deficiency may also compromise the proliferation and differentiation of erythroid progenitor cells [84]. A cis-element in the erythropoietin receptor promoter of human and mouse genes was identified by mutation analysis. The authors conclude that activation of the erythropoietin receptor gene by Wt1 may represent an important mechanism in normal erythropoiesis.

Our findings suggest that transcription factors identified with potential binding sites in the β-locus by *in silico *analysis (Figure 6C) may have biological relevance to *HBG *and *HBB *gene regulation. We tested our hypothesis by analyzing two factors *KLF4 *(Figure 3B) and *KLF12*; in a recently published study [85]. By electrophoresis mobility shift assay, we demonstrated that *KLF4 *and *KLF12 *directly bind the γ-globin CACCC element. However, only *KLF4 *mediated positive regulation of γ-globin expression in cell lines and primary erythroid progenitors. Interestingly, the *in silico *studies only identified *KLF4 *binding in the γ-globin promoter (Additional file 8: Table S7).

Additional support for our experimental approach can be gained from *C/EBP *proteins which have been demonstrated to activate γ-globin through binding of the distal CCAAT box [67]; C/EBP compete for binding of the repressor protein CCAAT displacement protein [86]. In fact, this is the proposed mechanism for hereditary persistence of fetal hemoglobin due to a C/T mutation at -117 in the distal CCAAT box [87]. These findings provide evidence that other transcription factors involved in globin gene regulation can be identified in our system.

## Conclusions

The one-phase liquid culture system was used to define the erythroid transcriptome associated with the γ/β-globin gene switch *in vitro *and to address global mechanisms involved in globin gene regulation. We utilized primary erythroid cells to characterize three major gene expression patterns including profile-1 genes silenced from day 7 to day 28, profile-2 genes activation from day 7 to day 28 and profile-3 genes with peak expression at day 21 around the time of the γ/β-globin switch. After erythroid differentiation the transcriptome was established using the Illumina BeadChip microarray platform. Profile-1 genes were related to hemopoeisis and the NF-Kβ, Interleukin-4 and p38 MAPK signaling pathways. Profile-2 genes were shown to be associated with cell proliferation, heme synthesis and erythrocyte differentiation. Finally profile-3 genes were associated with nucleotide metabolism, and protein switching. The biological subgroups generated by our data corroborate published studies related to erythroid maturation and hemoglobin synthesis during terminal differentiation. New insights into the switching process will be obtained by further investigation of biological subgroups associate with the γ/β switch observed at day 21 in culture.

## Methods

### Primary erythroid culture system

Peripheral blood mononuclear cells were isolated from three normal donors (Carter Blood Center, Bedford, TX) using Histapaque-1.077 (Cellgro Inc.). The mononuclear cells were grown in three independent cultures using the one-phase protocol as previously published [18]. Briefly, cells were cultured in αMEM containing 30% fetal bovine serum (Atlanta Biologicals, Atlanta GA), 1% deionized BSA with penicillin (100 U/mL) and streptomycin (0.1 mg/mL) at 37°C and 5% CO_2_. The following growth factors were added on day 0: stem cell factor (50 ng/mL), Interleukin-3 (10 ng/mL) and erythropoietin (4 U/mL). See details of our one-phase culture protocol in Additional file 11: Supplemental Methods. Approximately 1.5 million cells were harvested every 2-3 days from each culture for RNA isolation and morphological studies by cytospin preparations fixed in 100% methanol and stained with Giemsa. Cell counts and morphology were performed on a light microscope under oil emersion; at least 500 cells were counted on each slide.

### RT-qPCR analysis

The mRNA levels of γ-globin, β-globin, and glyceraldehyde-3-phosphate dehydrogenase (GAPDH) were measured as previously published [52,53,85] to establish the γ/β globin switch in culture. Total RNA was extracted from 5 × 10^6 ^cells at each time point from the three independent cultures using a Trizol kit (Invitrogen, Carlsbad, CA) according to the manufacturer's instructions. RNA qualities were confirmed by Bio-Rad *Experion *automated electrophoresis. Based on published criteria all samples used in our analysis had RQI values > 8.0. cDNA was prepared from total RNA (1 μg) using the Improm-II reverse transcriptase (RT) system (Promega, Madison, WI). The γ-globin, β-globin, and GAPDH mRNA levels were quantified by qPCR (iCycler iQ; Bio-Rad, Hercules, CA) and standard curves were generated using a Topo7-based plasmid carrying the γ-globin (Topo7- β-globin), Topo7- β-globin, or Topo7-GAPD cDNA.

### Illumina BeadChip microarray analysis

Total RNA isolated on day 7, 14, 21 and 28 was used for microarray analysis on the whole-genome Illumina HumanWG-6V2 Expression BeadChip with 48,700 probes (Illumina, Inc., San Diego, CA) per the manufacturer's protocol. Briefly, RNA samples (0.5 μg) were amplified using Illumina TotalPrep RNA amplification kit (Enzo, Austin, TX) with biotin UTP labeling. Single stranded cDNA was generated using a T7 oligo(dT) primer followed by second strand synthesis to generate double-stranded cDNA which was column purified. Biotin-labeled cRNA was synthesized by *in vitro *transcription using T7 RNA polymerase which was column purified, and checked for quality using the Bio-Rad Experion system (Hercules, CA). cRNA (1.5 μg) was hybridized to the Illumina BeadChip per protocol and streptavidin-Cy3 (Amersham, Piscataway, NJ) was used for detection. Chips were scanned on an Illumina Beadstation.

The following RT-qPCR approach confirmed the microarray data. Total RNA was isolated using the Trizol kit (Invitrogen) according to the manufacturer's instructions. RNA solutions were treated with DNase I before RT then cDNA was synthesized in a reaction containing SuperScript III RT (Invitrogen) and random hexamer primers. Gene specific primers were designed using Primer3 software http://frodo.wi.mit.edu/primer3/. PCR reactions were performed using a SYBR PCR master mix kit (AB Biosystems, Inc. Carlsbad, CA), and a Chromo4 Fluorescence Detector (Bio-Rad). The PCR protocol included denaturation at 95°C for 10 min, followed by 40 cycles of 95°C for 15 s and 60°C for 1 min and 18S or GAPDH RNA were used as internal controls. Serial dilutions of cDNAs generated from reference RNA (Strategene Inc., La Jolla, CA) were used to establish standard curves for each gene. The qPCR results were analyzed using Opticon software (Bio-Rad).

### Bioinformatics and biostatistics analysis

The raw data obtained from the scanner were summarized as probe level signal intensities using Illumina BeadStudio v2.1.3 (Illumina), then background subtraction and quantile normalization (Additional file 1: Figure S4A) were completed using the MBCB algorithm [88]. After data normalization, we performed time-course analysis with PCA software (NIA Array Analysis Tool http://lgsun.grc.nia.nih.gov/ANOVA/bin/login.cgi) to characterize three major genomic profiles [89]. For PCA, the genomics data were mined to follow the expression patterns of γ- and β- globin during erythropoiesis which is best analyzed based on the F-distribution with ANOVA = 0.01 and FDR = 0.05. Because we wanted to maintain globin gene profiles during our evaluation, permutation analysis was not conducted. The different profiles were analyzed at the > 1.25-fold, > 1.5-fold, and > 2.0-fold change levels in a time-course manner on day 7, 14, 21 and 28 to define three major gene profiles. For subsequent bioinformatics analyses, we used genes with > 1.5-fold change in expression (Additional file 6: Table S5).

To support the results of PCA, Hierarchical Clustering methods were also used. Profile-1, profile-2 and profile-3 with 1.25-fold, 1.5-fold, and 2.0-fold change were input into BRB ArrayTools http://linus.nci.nih.gov/BRB-ArrayTools.html[90]. After log-transformation the intensity values were loaded into the Gene Cluster 3.0, BRB platform. The various genes (rows) and culture times (columns) were clustered using the correlation distances with similarity metric and average linkage. Finally, a heat-map was generated using the average gene expression fold-change values at the different time points to demonstrate up-regulated genes (red), gene silencing (green) and day 7 normalized to one or no change in gene expression (black). Three major genomic profiles including profile-1 (gene silencing from day 7-28), profile-2 (gene activation from day 7-28) and profile-3 with peak expression at day 21 were generated. These data are available through the National Center for Biotechnology Information Gene Expression Omnibus [91] using accession number ID GSE35102.

To validate the microarray data we chose a subset of genes to analyze by RT-qPCR and performed statistical analysis to determine the correlation between microarray and RT-qPCR data. Subsets of profile-1 and profile-2 genes were analyzed using the correlation coefficient, R^2 ^and confidence intervals of parameter were also generated in the statistical analysis. Descriptive statistics for microarray and RT-qPCR data including raw counts, means and standard error were used to present the distribution of the measured parameters. Statistical assessment of these characteristics at different time points for microarray was performed using the Student's *t*-test (*p *< 0.05 was considered significant).

### DAVID GO analysis

DAVID GO software http://david.niaid.nih.gov was used to classify the large number of genes mined into biological functional groups. The gene subgroups were loaded into the GO platform and then a) the data were analyzed and Kappa scores calculated to evaluate the mining process; b) the transcriptome of each profile was categorized by biological process and molecular function using an over-representation analysis.

### Functional genomics analysis

The pathway analyses from the mined genes were performed using IPA (IPA Ingenuity Systems, Inc., Redwood City, CA) software. Core analysis was processed using direct and indirect relationships for pathway scoring. Profile-1, profile-2, and profile-3 genes were investigated using the IPA software according to the manufacturer's instruction. The final data were reported by Pathway map and "txt" version with a hit list; P-values < 1.3 log (-) was used as a cut off for statistical significance [91].

### Transcription factor analysis

To search for putative transcription factor binding sites in the β-globin locus on chromosome 11 the following software programs were used: TESS (http://www.cbil.upenn.edu/cgi-bin/tess/tess[92], TFSEARCH http://mbs.cbrc.jp/research/db/TFSEARCH.html) [93], FIRE http://quantbio-tools.princeton.edu/cgi-bin/FIRE/form.pl[94] and Weeder H version 1.0 [95]. The Genome Browser http://genome.ucsc.edu/ was employed to confirm motif coordinates in the Human Genome (version Hg 19). DNA sequences from the LCR, the 5' upstream *HBG1 *region, coding sequence for *HBG1 *and *HBG2*, and 3' downstream sequence (Figure 6A) were analyzed with the four platforms to identify putative binding sites for profile-1 genes. A second analysis of the LCR, *HBB *5' upstream, coding and 3' downstream regions was completed to identify putative binding sites for profile-2 genes. Potential transcription factor binding sites for profile-3 genes were analyzed in a combination of the regions tested for profile-1 and -2 genes.

## Abbreviations

ATM: Ataxia telangiectasia mutated gene; C/EBPA: CCAAT/enhancer-binding protein alpha; DBP: D site of albumin promoter binding protein; (HSs): DNaseI hypersensitive sites; ERK/MAPK: Extracellular signal-regulated kinase/mitogen-activated protein kinase; FOXM1: Forkhead box M1; FOXQ1: Forkhead box Q1; GATA1: GATA binding protein 1; GO: Gene ontology; IPA: Ingenuity pathway analysis; KLF1: Kruppel-like factor 1; (LCR): Locus control region; MBCB: Model-Based Background Correction for BeadArrays; NFE2: Nuclear factor erythroid 2; p38 MAPK: P38 mitogen activated protein kinase; PCA: Principal component analysis; STAT1: Signal transducer and activator of transcription 1; TESS: Transcription elements search system; TFSEARCH: Transcription Factor Search.

## Competing interests

The authors declare that they have no competing interests.

## Authors' contributions

BP and MS conceived, designed and guided the study and edited the manuscript. WL performed experiments including cell culture and drug inductions, and RNA specimen preparation. BL performed the bioinformatics analysis and drafted the manuscript. LD carried out the microarray processing and RT-qPCR confirmation and assisted with bioinformatics analysis. All authors read and approved the final manuscript.

## Supplementary Material

Additional file 1 **Figure S1**. RNA quality check. **Figure S4A **Multiple regression analysis. **Figure S5C **Mitotic Roles of Polo-Like Kinasae signaling pathway. **Figure S5D **ATM signaling pathway. Click here for file

Additional file 2 **Table S1**. Day 7 Illumina HumanWG-6V2 Expression BeadChip raw data. This file contains a table listing microarray raw data including the mean (M) and standard error (SEM) for each gene calculated from triplicate RNA samples extracted on day 7 from cells grown in the one-phase liquid culture system. Briefly, total RNA with amplified and biotin-labeled and then used for hybridization to the Illumina HumanWG-6V2 Expression BeadChip. Microarray raw data were generated on the Illumina Beadstation scanner. Click here for file

Additional file 3 **Table S2**. Day 14 Illumina HumanWG-6V2 Expression BeadChip raw data. This file contains a table listing microarray raw data including the mean (M) and standard error (SE) for each gene calculated from triplicate RNA samples extracted on day 14 from cells cultured in the one-phase liquid culture system. Briefly, total RNA with amplified and biotin-labeled and then used for hybridization to the Illumina HumanWG-6V2 Expression BeadChip. Microarray raw data were generated on the Illumina Beadstation scanner. Click here for file

Additional file 4 **Table S3**. Day 21 Illumina HumanWG-6V2 Expression BeadChip raw data. This file contains a table listing microarray raw data including the mean (M) and standard error (SE) for each gene calculated from triplicate RNA samples extracted on day 21 from cells cultured in the one-phase liquid culture system. Briefly, total RNA with amplified and biotin-labeled and then used for hybridization to the Illumina HumanWG-6V2 Expression BeadChip. Microarray raw data were generated on the Illumina Beadstation scanner. Click here for file

Additional file 5 **Table S4**. Day 28 Illumina HumanWG-6V2 Expression BeadChip raw data. This file contains a table listing microarray raw data including the mean (M) and standard error (SE) for each gene calculated from triplicate RNA samples extracted on day 28 from cells cultured in the one-phase liquid culture system. Briefly, total RNA with amplified and biotin-labeled and then used for hybridization to the Illumina HumanWG-6V2 Expression BeadChip. Microarray raw data were generated on the Illumina Beadstation scanner. Click here for file

Additional file 6 **Table S5**. Differential gene expression generated by PCA. This file contains three tables listing genes with > 1.5-fold change in expression during erythroid maturation in our one-phase liquid culture system. PCA data show the log change; a positive value indicates decreased gene expression from day 7 to day 28 in contrast to increased gene expression indicated by a negative log change. The correlation score indicates the level of agreement between the specific globin gene profile curve and PCA values. Click here for file

Additional file 7 **Table S6**. Gene Ontology analysis from profile-1, -2 and -3 gene subsets. This file contains a complete list of the DAVID GO terms generated for the three gene groups tested; the count number indicates how many genes were identified in the specific pathway by microarray analysis. The p values indicate the significance level for the specific pathways identified. The Benjamin score represents the adjusted multiple p values to take into account all genes involved in the pathways identified. Click here for file

Additional file 8 **Table S7**. TESS, TFSEARCH, Weeder H and Fire analysis for profile-1 genes. Click here for file

Additional file 9 **Table S8**. TESS, TFSEARCH, Weeder H and Fire analysis for profile-2 genes. Click here for file

Additional file 10 **Table S9**. TESS, TFSEARCH, Weeder H and Fire analysis for profile-3 genes. Click here for file

Additional file 11 **Supplemental Methods**. Method for the one-phase tissue culture system. Click here for file
