# Supplementary material for: Characterization of the transcriptome profiles related to globin gene switching during in vitro erythroid maturation
Source: BMC Genomics. 2012 Apr 26;13:153. doi: 10.1186/1471-2164-13-153 (PMC3353202; doi:10.1186/1471-2164-13-153)
Supplement: Additional file 1 — Figure S1. RNA quality check. Figure S4A Multiple regression analysis. Figure S5C Mitotic Roles of Polo-Like Kinasae signaling pathway. Figure S5D ATM signaling pathway. [file 1471-2164-13-153-S1.PPTX]

## Slide 1
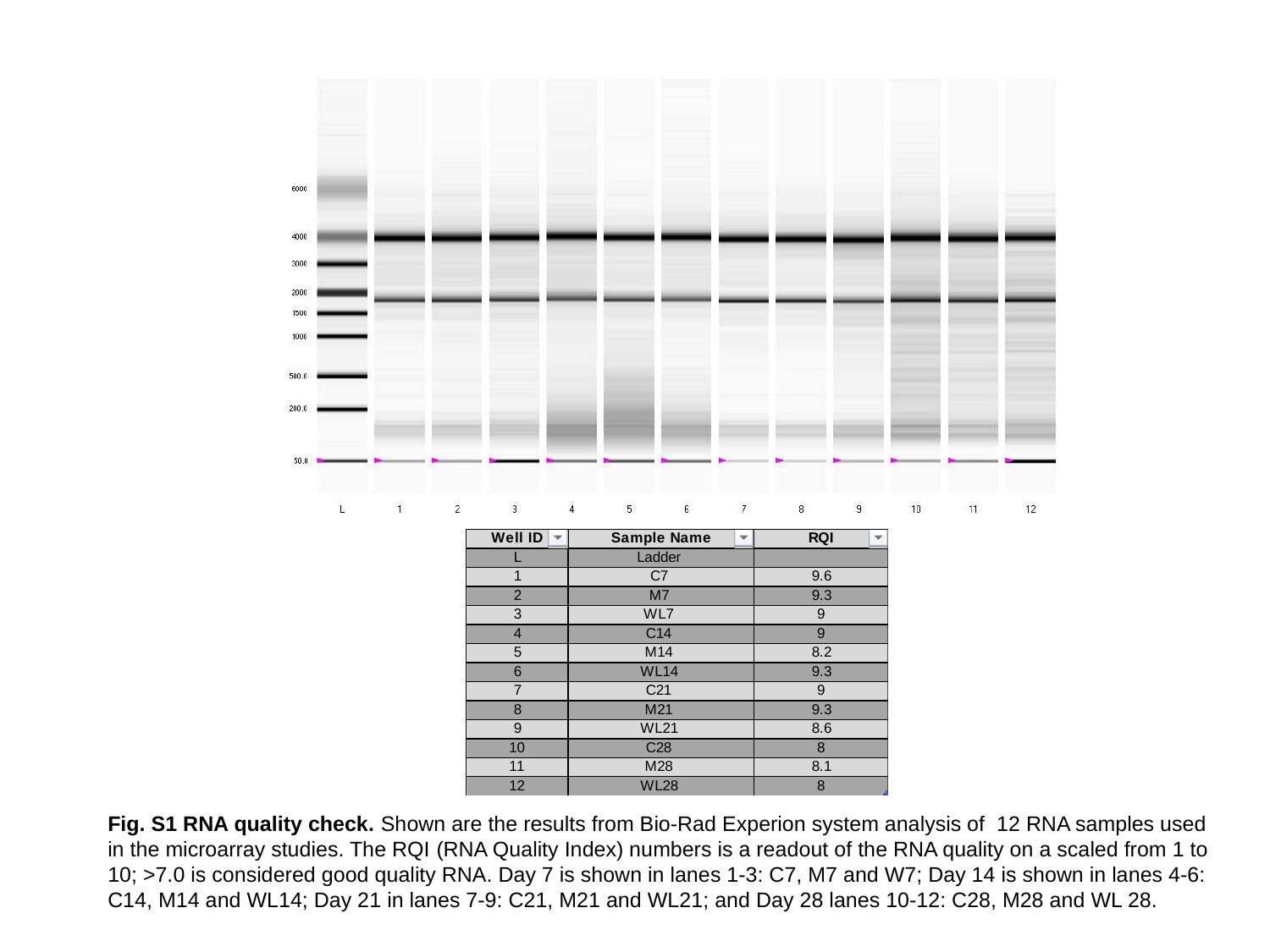

Fig. S1 RNA quality check. Shown are the results from Bio-Rad Experion system analysis of 12 RNA samples used in the microarray studies. The RQI (RNA Quality Index) numbers is a readout of the RNA quality on a scaled from 1 to 10; >7.0 is considered good quality RNA. Day 7 is shown in lanes 1-3: C7, M7 and W7; Day 14 is shown in lanes 4-6: C14, M14 and WL14; Day 21 in lanes 7-9: C21, M21 and WL21; and Day 28 lanes 10-12: C28, M28 and WL 28.

## Slide 2
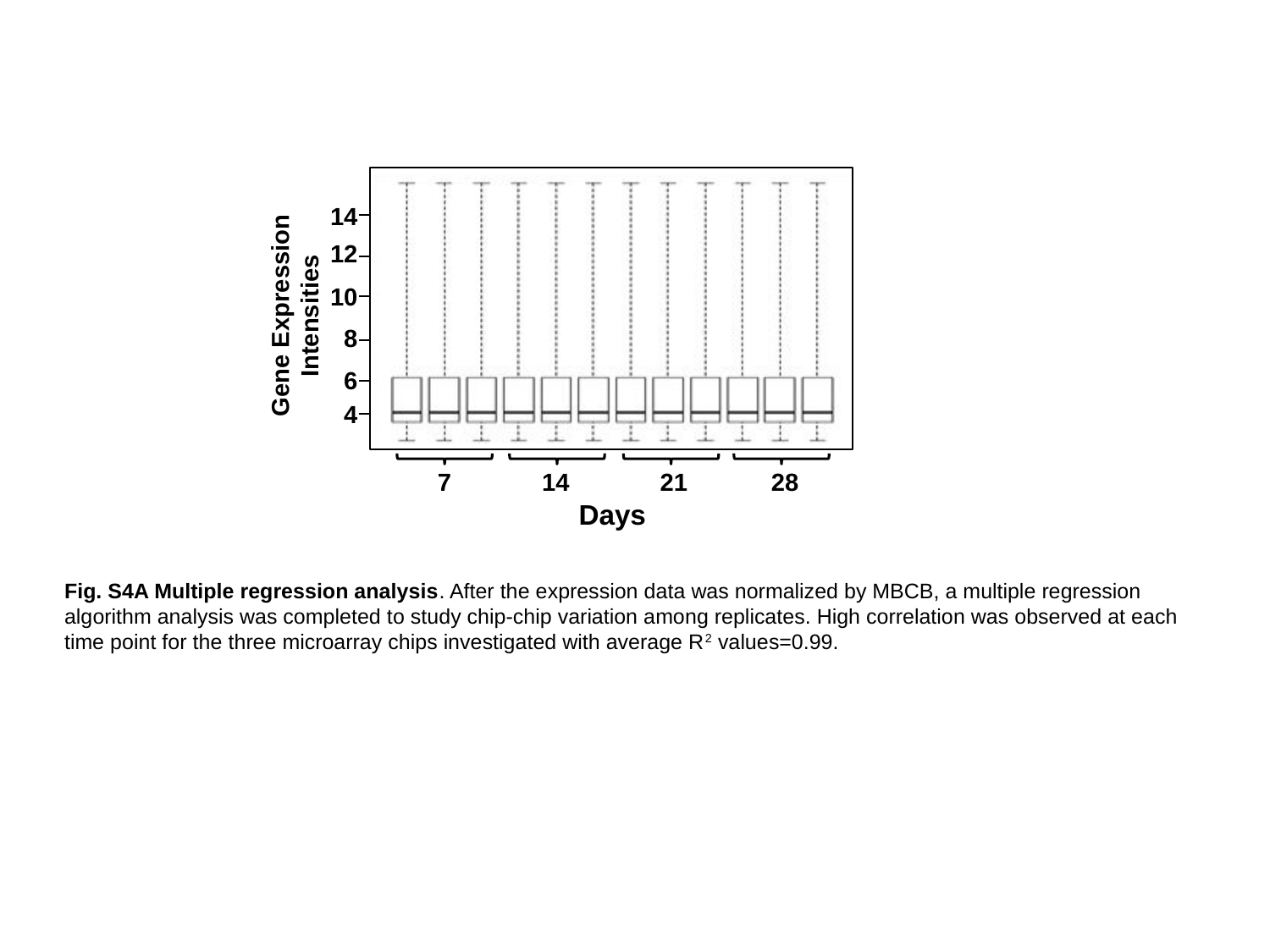

14
12
10
8
6
4
Gene Expression Intensities
 7 14 21 28
Days
Fig. S4A Multiple regression analysis. After the expression data was normalized by MBCB, a multiple regression algorithm analysis was completed to study chip-chip variation among replicates. High correlation was observed at each time point for the three microarray chips investigated with average R2 values=0.99.

## Slide 3
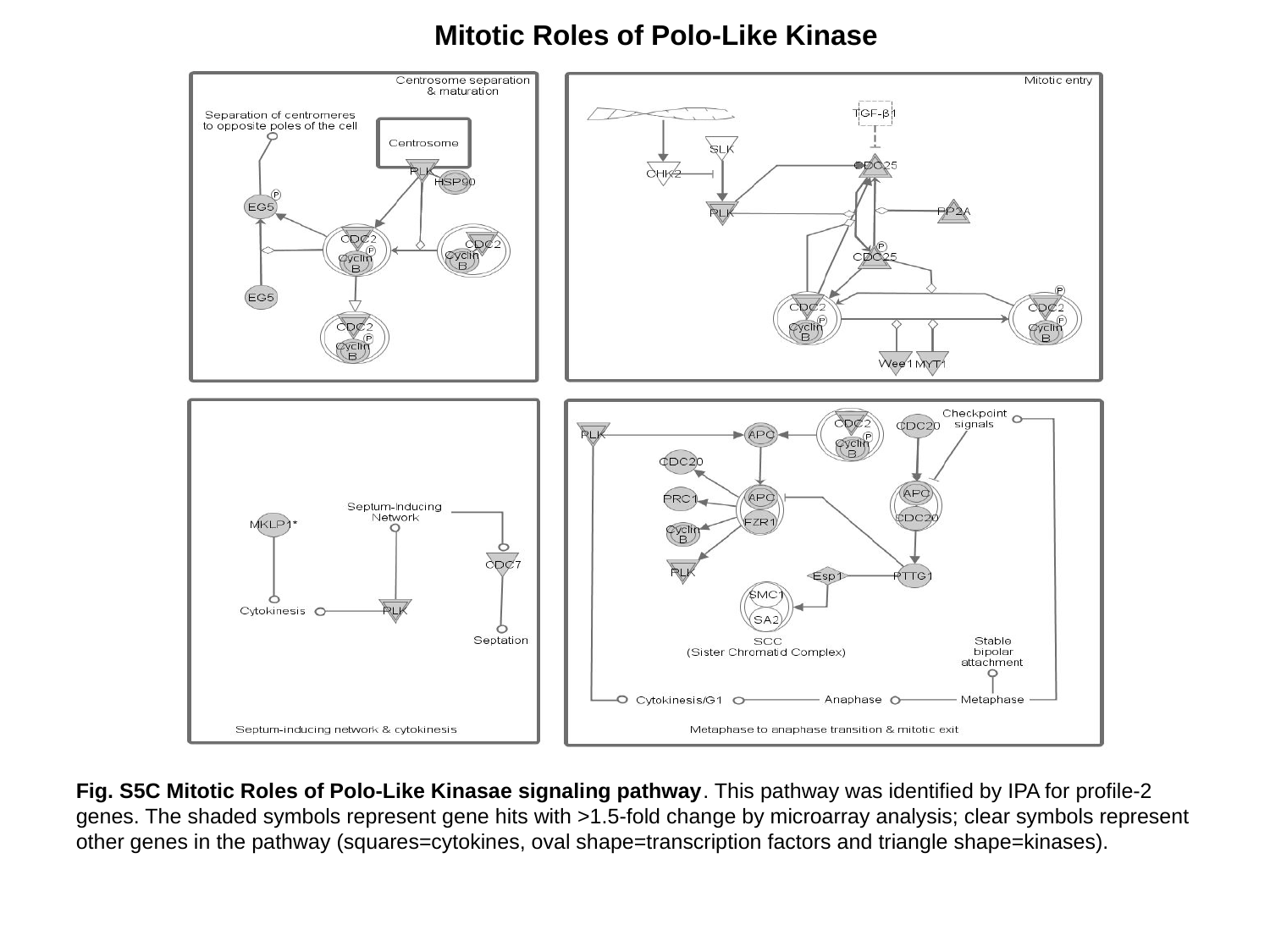

Mitotic Roles of Polo-Like Kinase
Fig. S5C Mitotic Roles of Polo-Like Kinasae signaling pathway. This pathway was identified by IPA for profile-2 genes. The shaded symbols represent gene hits with >1.5-fold change by microarray analysis; clear symbols represent other genes in the pathway (squares=cytokines, oval shape=transcription factors and triangle shape=kinases).

## Slide 4
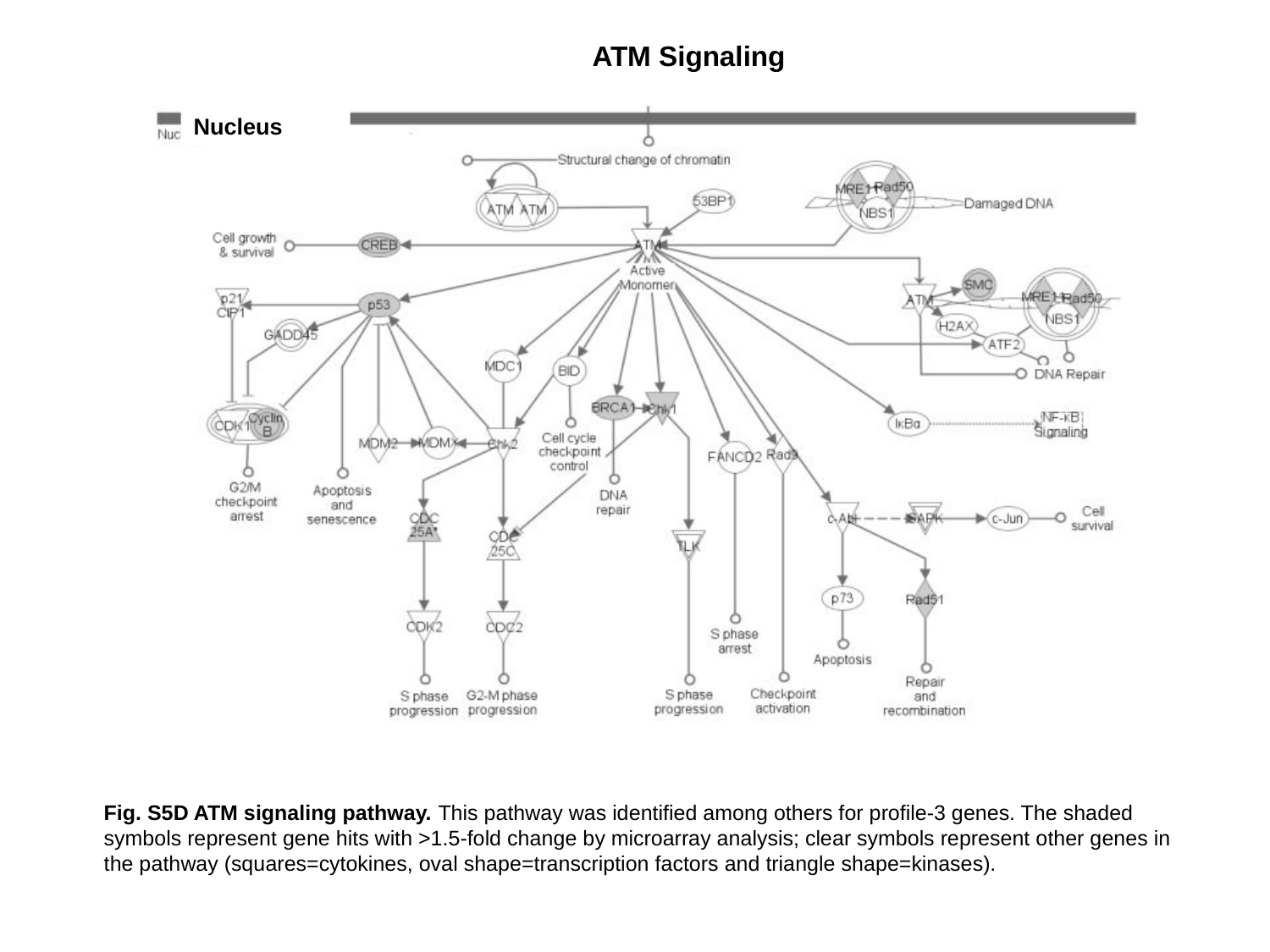

ATM Signaling
Nucleus
Fig. S5D ATM signaling pathway. This pathway was identified among others for profile-3 genes. The shaded symbols represent gene hits with >1.5-fold change by microarray analysis; clear symbols represent other genes in the pathway (squares=cytokines, oval shape=transcription factors and triangle shape=kinases).
