# Supplementary material for: Characterization of the transcriptome profiles related to globin gene switching during in vitro erythroid maturation
Source: BMC Genomics. 2012 Apr 26;13:153. doi: 10.1186/1471-2164-13-153 (PMC3353202; doi:10.1186/1471-2164-13-153)
Supplement: Additional file 8 — Table S7. TESS, TFSEARCH, Weeder H and Fire analysis for profile-1 genes. [file 1471-2164-13-153-S8.DOCX]

**Table S7** **TESS, TFSEARCH, Weeder H and Fire analysis for profile-1 genes**

| Symbol | Microarray (fold change) | | | |  | | Transcription factor binding motif | | |  |
| --- | --- | --- | --- | --- | --- | --- | --- | --- | --- | --- |
|  | Day 7 | Day 14 | Day 21 | Day 28 | ^1^Genomic location | ^2^Binding motif^ | Log-likelihood scores | ^3^P-value | ^4^β-Locus position | Gene location |
| *IKZF1* | 1 | 0.59 | 0.36 | 0.42 | 5310096 | TTGATATT | 10.14 | 0.038 | 8722-8729 | 5’HS4 |
| *RBPJ* | 1 | 0.65 | 0.51 | 0.23 | 5310376 | CAYGTGAY(R) | 14 | 0.000 | 11867-11874 | 5’HS3 |
| *POU5F1* | 1 | 0.78 | 0.62 | 0.53 | 5306198 | ACAGC (R) | 8.5 | 0.000 | 12627-12631 | HS3 |
| *ZEB1* | 1 | 0.74 | 0.42 | 0.45 | 5305969 | CAGAAACAT (R) | 18 | 0.000 | 14852-14860 | 3’HS3 |
| *NFKB1* | 1 | 0.65 | 0.38 | 0.21 | 5303971 | TAATCCC | 8.17 | 0.056 | 16364-16370 | 5’HS2 |
| *STAT1* | 1 | 0.63 | 0.13 | 0.28 | 5302295 | GTTTTATT | 16 | 0.000 | 16528-16535 | 5’HS2 |
| *LEF1* | 1 | 0.95 | 0.14 | 0.08 | 5302238 | CAGGTG (R) | 9.67 | 0.000 | 16579-16592 | 5’HS2 |
| *STAT2* | 1 | 0.83 | 0.3 | 0.57 | 5302072 | GTTCT (R) | 7.38 | 0.000 | 16750-16754 | HS2 |
| *DBP* | 1 | 1.13 | 0.28 | 0.67 | 5301626 | AGCGGCAG | 10 | 0.000 | 17204-17211 | 3’HS2 |
| *TCF7L2* | 1 | 0.45 | 0.15 | 0.17 | 5300716 | AGAAAGGAA | 13.71 | 0.031 | 18110-18119 | 3’HS2 |
| *ETS2* | 1 | 1.06 | 0.5 | 0.24 | 5279954 | RTCACRTG | 14 | 0.000 | −4011 to −4017 | 5’Gγ-globin |
| *KLF4* | 1 | 2.01 | 0.52 | 0.46 | 5272732 | CCYYTYYYTYNTTY | 19 | 0.051 | −1713 to −1725 | 5’Gγ-globin |
| *C/EBPB* | 1 | 0.9 | 0.15 | 0.48 | 5276636 | MTTNCNNMA (R) | 13 | 0.000 | −674 to −683 | 5’Gγ-globin |
| *C/EBPD* | 1 | 1.13 | 0.15 | 0.29 | 5276636 | MTTNCNNMA (R) | 10 | 0.000 | −674 to −683 | 5’Gγ-globin |
| *C/EBPA* | 1 | 1.2 | 0.09 | 0.1 | 5276503 | ATTTGGAAA | 12 | 0.000 | −559 to −568 | 5’Gγ-globin |
| *FOS* | 1 | 1.47 | 0.07 | 0.27 | 5276799 | GTCA (R) | 8 | 0.000 | −517 to −520 | 5’Gγ-globin |
| *GATA2* | 1 | 1.87 | 0.95 | 0.59 | 5271226 | AACGG | 12 | 0.041 | −160 to −164 | 5’Gγ-globin |
| *RUNX2* | 1 | 1.2 | 0.26 | 0.23 | 5271226 | AACGG | 10 | 0.041 | −160 to −164 | 5’Gγ-globin |
| *GATA2* | 1 | 1.87 | 0.95 | 0.59 | 5271203 | CCATG | 12 | 0.000 | −137 to −141 | 5’Gγ-globin |
| *NR3C1* | 1 | 0.87 | 0.44 | 0.4 | 5274886 | GAGAGCG | 10.49 | 0.210 | 43941-43947 | Gγ-globin IVSII |
| *MAF* | 1 | 1.32 | 0.21 | 0.22 | 5271885 | CGCCAAA (R) | 14 | 0.000 | −859 to −863 | 5'Aγ-globin |
| *NFIL3* | 1 | 0.75 | 0.23 | 0.19 | 5271708 | RTKAYGTAAY | 16 | 0.000 | −689 to −698 | 5’Aγ-globin |
| *USF2* | 1 | 1.18 | 0.4 | 0.34 | 5272173 | ACTTCC (R) | 10.23 | 0.600 | −1161 to −1166 | 5’Gγ/Aγ-globin |
| *GATA3* | 1 | 0.77 | 0.07 | 0.04 | 5271036 | GTTACTA (R) | 9.85 | 0.062 | −23 to −29 | 5’Gγ/Aγ-globin |

^1^Genomic coordinator locations shown begin from the motif located on Chromosome 11, version Hg19

^2^Binding motif is plus sequence except for R, minus sequence motif

^3^Approximate p-value for log-likelihood scores

^4^The negative numbers indicate position relative to the globin gene cap site

Abbreviations: Y, pyrimidine such as thymine or cytosine; M, adenine or cytosine; N, guanine or adenine or thymine or cytosine; K, G or T; R, purine such as adenine or guanine
