# Supplementary material for: Characterization of the transcriptome profiles related to globin gene switching during in vitro erythroid maturation
Source: BMC Genomics. 2012 Apr 26;13:153. doi: 10.1186/1471-2164-13-153 (PMC3353202; doi:10.1186/1471-2164-13-153)
Supplement: Additional file 9 — Table S8. TESS, TFSEARCH, Weeder H and Fire analysis for profile-2 genes. [file 1471-2164-13-153-S9.DOCX]

**Table S8** **TESS, TFSEARCH, Weeder H and Fire analysis for profile-2 genes**

| Symbol | Microarray (fold change) | | | |  |  | Transcription factor binding motif | | | |
| --- | --- | --- | --- | --- | --- | --- | --- | --- | --- | --- |
|  | Day 7 | Day 14 | Day 21 | Day 28 | ^1^Genomic location | ^2^Binding motif | Log-likelihood scores | ^3^P-value | ^4^β-Locus position | Gene location |
| *TCF3* | 1 | 0.93 | 2.4 | 4.43 | 5313535 | CTTTGAT (R) | 14 | 0.000 | 2584-2590 | 5’HS5 |
| *KLF10* | 1 | 0.83 | 1.19 | 1.77 | 5309431 | GGTGG | 7.65 | 0.000 | 9391-9396 | HS4 |
| *FOXM1* | 1 | 0.47 | 8.2 | 9.56 | 5299736 | AAAACAAACAAA | 24 | 0.000 | 19076-19088 | 3’HS2 |
| *NFIC* | 1 | 1.44 | 2.3 | 2.5 | 5304006 | CATTGGC (R) | 14 | 0.000 | 20012-20018 | 3’HS2 |
| *NFE2* | 1 | 1.53 | 10.98 | 17.47 | 5301976 | TGCTGAGTCAT | 17.25 | 0.001 | 16844-16854 | HS2 |
| *GATA1* | 1 | 1.83 | 94.63 | 112.29 | 5248468 | WGATAR | 10 | 0.000 | −246 to −252 | 5'β-globin |
| *KLF1* | 1 | 1.75 | 80.72 | 108.48 | 5248366 | CCACACCC | 14.37 | 0.014 | −136 to −143 | 5'β-globin |

^1^Genomic coordinator locations shown begin from the motif located on Chromosome 11, version Hg19

^2^Binding motif is plus sequence except for R, minus sequence motif

^3^Approximate p-value for log-likelihood scores

^4^The negative numbers indicate position relative to the globin gene cap site

Abbreviations: Y, pyrimidine such as thymine or cytosine; M, adenine or cytosine; N, guanine or adenine or thymine or cytosine; K, G or T; R, purine such as adenine or guanine
