# Supplementary material for: Characterization of the transcriptome profiles related to globin gene switching during in vitro erythroid maturation
Source: BMC Genomics. 2012 Apr 26;13:153. doi: 10.1186/1471-2164-13-153 (PMC3353202; doi:10.1186/1471-2164-13-153)
Supplement: Additional file 10 — Table S9. TESS, TFSEARCH, Weeder H and Fire analysis for profile-3 genes. [file 1471-2164-13-153-S10.DOCX]

**Table S9 TESS, TFSEARCH, Weeder H and Fire analysis for profile-3 genes**

| Symbol | Microarray (fold change) | | | |  | | Transcription factor binding motif | | |  |
| --- | --- | --- | --- | --- | --- | --- | --- | --- | --- | --- |
|  | Day 7 | Day 14 | Day 21 | Day 28 | ^1^Genomic location | ^2^Binding Motif | Log-likelihood scores | ^3^P-value | ^4^β-Locus position | Gene location |
| *NME1* | 1 | 0.64 | 1.79 | 0.59 | 5298162 | ATGRATAW (R) | 14 | 0.000 | 20657-20664 | 5’HS1 |
| *NME2* | 1 | 0.91 | 1.18 | 0.54 | 5306044 | GGGTGGG | 14 | 0.000 | 12776-12782 | HS3 |
| *MYB* | 1 | 0.91 | 4.2 | 2.79 | 5248252 | PyAACTG | 12 | 0.000 | −29 to −34 | 5’β-globin |
| *GATA5* | 1 | 0.94 | 63.75 | 36.59 | 5248753 | WGATAR | 10 | 0.000 | −487 to −492 | 5'β-globin |
| *POU4F1* | 1 | 1.28 | 1.39 | 0.90 | 5271245 | AATGCAAAT (R) | 18 | 0.000 | −228 to −236 | 5’Gγ/Aγ-globin |
| *WT1* | 1 | 1.26 | 6.01 | 2.77 | 5311535 | CAGCCTCAC (R) | 18 | 0.000 | 7285-7293 | 3’HS5 |

^1^Genomic coordinator locations shown begin from the motif located on Chromosome 11, version Hg19

^2^Binding motif is plus sequence except for R, minus sequence motif

^3^Approximate p-value for log-likelihood scores

^4^The negative numbers indicate position relative to the globin gene cap site

Abbreviations: Y, pyrimidine such as thymine or cytosine; M, adenine or cytosine; N, guanine or adenine or thymine or cytosine; K, G or T; R, purine such as adenine or guanine
