# Supplementary material for: Characterization of the transcriptome profiles related to globin gene switching during in vitro erythroid maturation
Source: BMC Genomics. 2012 Apr 26;13:153. doi: 10.1186/1471-2164-13-153 (PMC3353202; doi:10.1186/1471-2164-13-153)
Supplement: Additional file 11 — Supplemental Methods. Method for the one-phase tissue culture system. [file 1471-2164-13-153-S11.DOCX]

**Supplemental Methods**

Peripheral blood mononuclear cells were isolated from the buffy coat of one unit of blood collected from normal donors (Carter Blood Center, Bedford, TX) and used to establish our one-phase liquid culture system.

**A. Peripheral Blood Mononuclear Cells (PBNC) Isolation**

1. Dilute PBNC 1:1 (V/V) with phosphate buffered saline (PBS), pH 7.4. Gently layer one part of diluted PBMC onto one part of lymphocyte separation medium (Histapaque-1.077; Cellgro Inc.); 50 ml per unit of PBMC

2. Centrifuge at 1,400 rpm for 30 min, at room temperature

3. Aspirate off the top layer (plasma + platelets)

4. Collect mononuclear cells (interphase layer) with a pipette and transfer to new 50 ml tubes

5. Add an equal volume of PBS, mix and centrifuge at 1,200 rpm for 5 min

6. Aspirate the supernatant and mix the pellet by tapping; wash twice with PBS; centrifuge at 1,000 rpm for 5 min after each wash

**B. One-Phase Medium (all growth factors were purchased from Sigma)**

1. Minimum Essential Medium Alpha (α-MEM)

2. Penicillin l00U/ml and streptomycin 0.lmg/ml

3. Warm the medium to 37ºC and then add:

-30% fetal bovine serum (**not heat-inactivated)**

-BSA 1% (**deionized)**

-Human recombinant erythropoietin (4U/mL)

-Stem cell factor (50ng/mL)

-Interleukin-3 (10 ng/mL)

-β-mercaptoethanol (10^−5^M)

-Dexamethasone sodium phosphate (10^−6^M)

-Human hollo-transferrin (0.3mg/mL)

**C. Tissue Culture set up**

1. Resuspend the cell pellet in One-Phase medium; adjust the medium volume to achieve a cell density of 6 million cells per ml
2. Incubate cells at 37ºC and 5% C0_2_ in an humidified incubator
3. On day 14 – pass cells in freshly prepared One-Phase medium
